# Effect-site concentration of propofol required for LMA-Supreme™ insertion with and without remifentanil: a randomized controlled trial

**DOI:** 10.1186/s12871-015-0115-8

**Published:** 2015-10-06

**Authors:** Matilde Zaballos, Emilia Bastida, Salomé Agustí, Maite Portas, Consuelo Jiménez, Maite López-Gil

**Affiliations:** 1 Department of Toxicology, Faculty of Medicine, Complutense University, Madrid, Spain; 2 Department of Anaesthesiology, Hospital Universitario Gregorio Marañón, Madrid, Spain; 3 Department of Anaesthesia, Head Department of Anaesthesiology, Hospital Universitario Gregorio Marañón, Madrid, Spain

**Keywords:** Anaesthetics, Intravenous, Propofol, Remifentanil, Equipment, Laryngeal mask airway supreme

## Abstract

**Background:**

A new supraglottic device, the LMA-Supreme™, has recently become available for clinical use. Information on anaesthetic and co-adjuvant requirements for insertion of the LMA-Supreme™ is limited. The present study aimed to evaluate the optimal effect-site concentration of propofol in 50 % (EC_50_) of adults necessary for successful insertion of the LMA-Supreme™ and to examine remifentanil’s effect on propofol requirements.

**Methods:**

Fifty-eight elective patients (aged 18–60 years; ASA (American Society Anaesthesiologists) physical status classification I and II) scheduled for day surgery were randomly assigned to one of two groups: propofol with saline or propofol with remifentanil. Anaesthesia was induced by target-controlled infusion according to predetermined effect-site concentrations of propofol and remifentanil (5 ng.mL^−1^). The EC_50_ was calculated using Dixon’s up-and-down method. Ten minutes following drug administration, LMA-Supreme™ insertion was attempted without the use of muscle relaxant drugs.

**Results:**

In the propofol + saline group, the EC_50_ of propofol required for LMA-Supreme™ insertion was 6.32 ± 0.67 μg.mL^−1^ (95 % CI, 5.69–6.94 μg.mL^−1^). With the addition of remifentanil at an effect-site concentration of 5 ng.mL^−1^, the EC_50_ of propofol required for LMA-Supreme™ insertion was 2.50 ± 0.80 μg.mL^−1^ (95 % CI, 1.82–3.17 μg.mL^−1^; *p* < 0.0001).

**Conclusions:**

The propofol requirement for smooth insertion of the LMA-Supreme™ was 60 % less when remifentanil (5 ng.mL^−1^) was co-administered.

**Clinical trial registration:**

Identified as NCT01974648 at www.clinicaltrials.gov.

## Background

The Laryngeal Mask Airway Supreme™ (LMA-Supreme™; Laryngeal Mask Company Limited, Singapore) is a new, single-use laryngeal mask airway that was recently introduced and combines some of the features of the LMA-Fastrach™ and the LMA-ProSeal™. The mask utilizes an airway tube with an elliptical cross section, which is intended to facilitate placement without requiring the insertion of fingers into the mouth. The LMA-Supreme™ incorporates a drainage tube to permit drainage from and access to the stomach and to provide confirmatory evidence of correct placement [1]. To minimize accidental rotation, the airway tube of the LMA-Supreme™ is much stiffer than that of the LMA-Classic™ or the LMA-ProSeal™. Recently, there has been a growing interest in this device because of favourable studies obtained in several anaesthetic contexts that have proven its effectiveness and safety [2, 3].

Propofol has been extensively used for insertion of supraglottic devices, generally providing optimal conditions. However, co-induction agents such as opioids are commonly used with propofol to facilitate device insertion and to reduce the dose of propofol along with the adverse effects associated with large propofol doses. When administered with propofol, the ultrashort-acting opioid remifentanil improves clinical conditions for insertion of supraglottic airways and reduces the concentration of propofol required for this purpose [4].

The propofol requirement for insertion of the LMA-Supreme™ is unknown. Most available data on anaesthetic and co-induction agent requirements for insertion of the LMA-Supreme™ originate from research focused on other evaluations of the device, such as the adequacy of ventilation achieved and the incidence of complications [2, 3, 5, 6].

Previous studies have shown that the anaesthetic requirements for insertion of different airway devices are dissimilar; these differences may be related to structural changes associated with these airway devices that exert different pressures and forces in the oral and pharyngeal cavities [7, 8]. Consequently, considering the structural features of the LMA-Supreme™ (a stiffer airway tube, similar to that of the LMA-Fastrach™; large surface area of the inflatable cuff), we postulated that these characteristics might influence the anaesthetic requirements for its insertion.

The aim of this randomized controlled study was to determine the optimal effect-site concentration of propofol in 50 % (EC_50_) of adults necessary for successful insertion of the LMA-Supreme™ and to evaluate whether remifentanil administration reduces the EC_50_. We hypothesized that remifentanil would diminish the effect-site concentration of propofol required for LMA-Supreme™ insertion.

## Methods

This study was a prospective, randomized, double-blind placebo-controlled trial (trial registry: NCT01974648 at www.clinicaltrials.gov). Study approval was obtained from the Institutional Review Board of Hospital Gregorio Marañón (Chairman Dr. F. Díaz Otero, dated May 3, 2011, code: FIBHGM-ECNC002-2011, Madrid, Spain); oral and written informed consent was obtained from the study participants. The study was conducted at Hospital Gregorio Marañón (Madrid, Spain) from May 2012 to June 2013.

The trial was carried out in accordance with the principles of the Helsinki Declarations. The CONSORT recommendations for reporting randomized, controlled trials were followed.

We enrolled ASA physical status I and II patients aged 18–60 years who were scheduled for general anaesthesia in our ambulatory surgery unit.

Patients with a potentially difficult airway (Mallampati III or IV, thyromental distance of less than 6 cm, a limited mouth opening and/or cervical spine disease, sleep apnoea syndrome), reactive airway disease, signs of upper respiratory infection, and those showing hiatus hernia or oesophageal reflux were excluded from the study.

The first author (MZ) enrolled and informed the participants, and the second author (EB), who was not involved in patient care, generated the allocation sequences using a computer-generated program. The eligible patients were randomly assigned to either the propofol + saline group or the propofol + remifentanil group by simple randomization.

The randomized sequence was stored in sealed opaque envelopes. If the patient fulfilled the inclusion criteria, the investigator (EB) opened the sealed envelope in the operating room. After opening the envelope, the corresponding group assignment was communicated to the attending anaesthesiologist.

Routine non-invasive monitoring (arterial blood pressure and heart rate), pulse oximetry, and a bispectral index (BIS) monitor (A- 2000™ version 3.4; Aspect Medical Systems Inc., Norwood, USA) were attached. The patients received 1 mg intravenous midazolam 20 min before induction of anaesthesia. Haemodynamic parameters were measured every minute.

The patients were pre-oxygenated with 100 % oxygen for 3 min. Target-controlled infusions (TCIs) of propofol and remifentanil were administered with commercial TCI pumps (Alaris® PK; Cardinal-Health, Rolle, Switzerland) according to the pharmacokinetic models of Schnider and Minto, respectively [9, 10].

Anaesthesia was induced using propofol (Diprivan prefilled syringes containing 1 % propofol; Zeneca Pharmaceuticals, Macclesfield, UK) at the predetermined concentration. Propofol infusion was coadministered with either saline or remifentanil at an effect-site concentration of 5 ng.mL^−1^. The first patient in each group received an effect-site concentration of propofol of 4.0 μg.mL^−1^.

Ten minutes following drug administration, a skilled anaesthetist (a staff member with more than 100 LMA uses) inserted the LMA-Supreme™. The LMA-Supreme™ was placed according to the manufacturer’s recommended insertion technique [1]. A size 4 LMA-Supreme™ was used for women and a size 5 for men; however, a size 3 was chosen for patients weighing ≤ 50 kg. After insertion, the cuff was inflated with air to a maximum pressure of 60 cm H_2_O using an aneroid barometer (Mallinckrodt™ Anesthesia; Tyco/Healthcare, USA). Neuromuscular blocking agents were not administered.

Two nurses, who were blinded to the propofol concentration, evaluated each patient’s response to LMA-Supreme™ insertion. They classified the response as either “movement” or “no movement”. Movement was defined as the presence of coughing, straining, bucking, or gross purposeful muscular movement within 1 min of airway insertion. The presence of these responses to LMA-Supreme insertion was considered as failure of insertion. Minor movement and hiccups were not considered to represent movement in this trial. The absence of movement to LMA-Supreme insertion was considered as success of insertion.

Patients experiencing movement received a 1 mg.kg^−1^ bolus of propofol.

Jaw relaxation was graded according to the previously reported Muzi score: 1 = fully relaxed, 2 = mild resistance, 3 = resistance but the jaw could be opened, and 4 = resistance requiring a dose of propofol [11]. Jaw relaxation and difficulty in mouth opening were judged solely by the investigator (MZ).

The propofol effect-site concentration used for each patient was determined by the response of the previous patient using the modified sequential “up-and-down method” described by Dixon [12]. Each patient’s response determined the concentration of propofol used for the next patient. The first patient received propofol at a 4.0 μg.mL^−1^ effect-site concentration, and the step size of increases/decreases was 0.5. μg.mL^−1^. If insertion of the LMA-Supreme™ was a success, the target propofol effect-site concentration for the next patient was set 0.5 μg.mL^−1^ lower than before. If insertion of the LMA-Supreme™ failed, the target propofol effect-site concentration for the next patient was set 0.5 μg.mL^−1^ higher than before. A single measurement was obtained from each patient, and the sequence was continued until a sample size of seven crossovers was reached in each group.

After stable ventilation with oxygen in air, oropharyngeal leak pressure (OLP) was measured as follows: the expiratory valve was closed and fresh gas flow to the patient was maintained at 3 L.min^−1^; the rising pressure within the system was measured with the pressure gauge and allowed to increase until it stopped rising (the expiratory valve was limited to 40 cm H_2_O) [13].

After the OLP was measured, a 3.5-mm fiberscope (Karl Storz GmbH & Co. KG, Tuttlingen, Germany) was introduced 1 cm proximal to the end of the airway tube, and the laryngeal view was recorded. The fiberscope score was classified using an established scoring system as follows: grade I = vocal cords not seen, II = vocal cords plus anterior epiglottis seen, III = vocal cords plus posterior epiglottis seen, and IV = only vocal cords seen [14].

Haemodynamic data were recorded at baseline (before induction of anaesthesia), immediately before LMA-Supreme™ insertion, and every minute after LMA-Supreme™ insertion for the first 6 min.

Hypotension, defined as a decrease in mean arterial pressure of more than 30 % compared with pre-induction values, and bradycardia, defined as a decrease in heart rate of more than 30 % compared with pre-induction values or a heart rate < 40 bpm, were treated with ephedrine 3 mg or atropine 0.01 mg.kg^−1^, respectively.

### Statistical analysis

Effect-site propofol concentrations were determined by calculating the midpoint concentration of all independent pairs of patients who manifested crossover from a movement response to a non-movement response. The EC_50_ of propofol was defined as the average of the crossover midpoints (mean) in each group. The standard deviation (SD) of the EC_50_ represented the SD of the crossover midpoints of each group. Predicted EC_50_ values in the propofol + saline and propofol + remifentanil groups were compared using Student’s *t*-test.

We also analysed our data using logistic regression curves to determine the probability of “no movement” relative to propofol concentration and to obtain propofol concentrations where 50 % (EC_50_) and 95 % (EC_95_) of the device insertion attempts were successful in both groups [15].

The airway leak pressures in the propofol + saline and propofol + remifentanil groups were compared using Student’s *t*-test.

Haemodynamic and BIS values were compared by repeated measures analysis of variance. The Chi-squared test, with Fisher’s exact probability test when appropriate, was used for comparing jaw relaxation and fibreoptic position.

A *p*-value of less than 0.05 was considered statistically significant.

All statistical analyses were performed with the SPSS-20 software package (IBM Corp., NY, USA).

### Sample size

Dixon methodology suggests that the experiment has to be continued until at least four crossovers are reached. In similar studies in the field of anaesthesia, the number of crossovers varies between six and eight, with six crossovers being most common. For this study’s purposes, seven crossovers were considered sufficient to identify the EC_50_ of propofol required to insert the LMA-Supreme™ [12, 16].

## Results

**Fig. 1:**
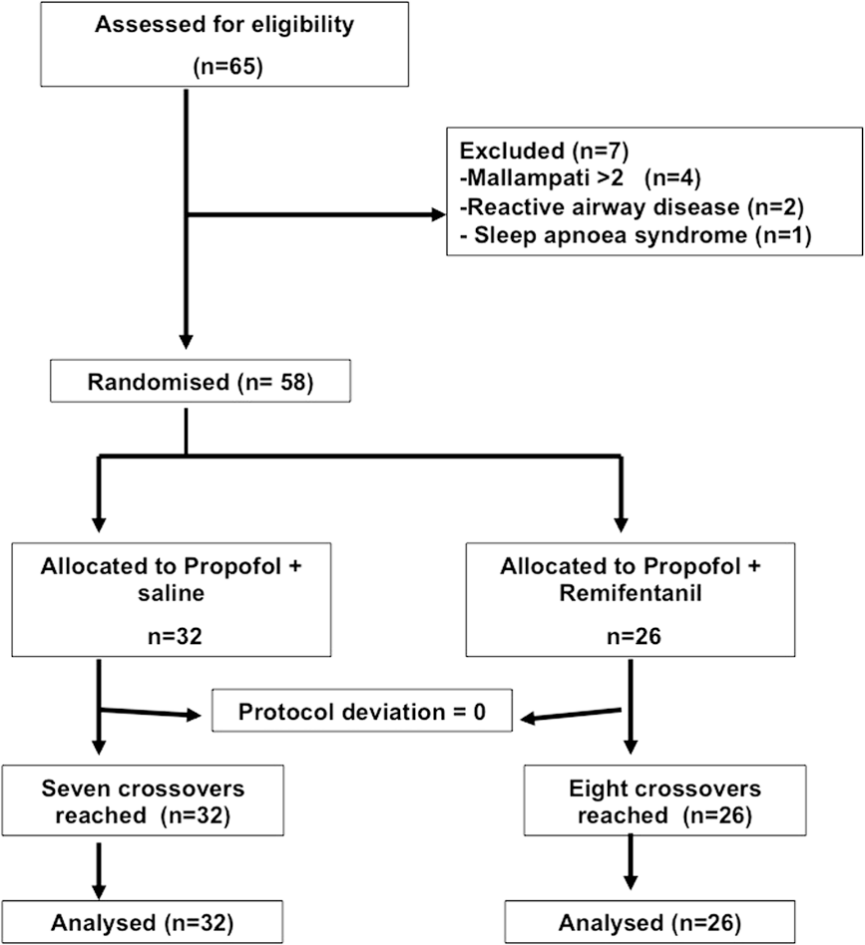
Flow-diagram of patient progress through the phases of the trial. Patients were recruited until a sample size of seven crossovers was reached in each group

A total of seventy patients were assessed for eligibility (Fig. 1). Of the 65 patients screened, 7 patients were excluded. Ultimately, 58 patients were randomized and received their allocated intervention. There were no significant differences in patient demographics or scheduled surgeries between the groups (Table 1).

**Table 1:** Demographic data and surgical procedures

	Propofol + saline	Propofol + remifentanil
Patients	32	26
Age (yr)	44 (9)	45 (12)
Female/Male	18/14	14/12
Weight (kg)	78 (13)	77 (14)
Height (cm)	169 (10)	168 (10)
BMI (kg.m^−2^)	27 (4)	27 (3)
Mallampati		
I	19	17
II	13	9
ASA I/ASA II	23/9	15/11
Surgical procedure		
Vascular (varicose vein surgery)	22	21
Orthopaedic	5	5
General surgery	5	0

Data are expressed as mean (SD) or number

*ASA* American society of anesthesiologists physical status, *BMI* body mass index, *SD* standard deviation

**Fig. 2:**
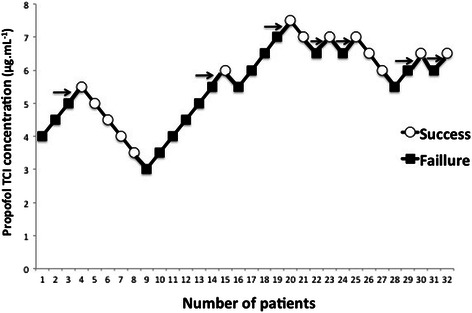
Patients’ responses to Laryngeal Mask Airway Supreme™ insertion in the propofol + saline group. Arrows indicate the midpoint of the effect-site concentration of all independent pairs of patients involving crossover from device insertion failure to successful Laryngeal Mask Airway Supreme™ insertion

**Fig. 3:**
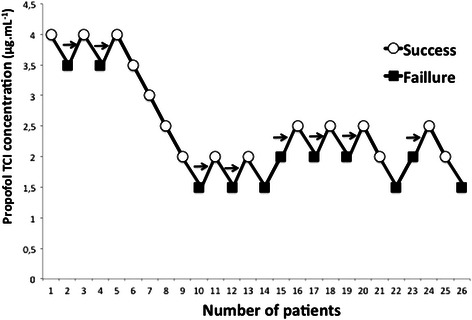
Patients’ responses to Laryngeal Mask Airway Supreme™ insertion in the propofol + remifentanil group. Arrows indicate the midpoint of the effect-site concentration of all independent pairs of patients involving crossover from device insertion failure to successful Laryngeal Mask Airway Supreme™ insertion

Dose–response data obtained using the up-and-down method are shown in Figs. 2 and 3.

In the propofol + saline group, the EC_50_ of propofol required for LMA-Supreme™ insertion was 6.32 ± 0.67 μg.mL^−1^ (95 % CI, 5.69–6.94 μg.mL^−1^). With the addition of 5 ng.mL^−1^ of remifentanil, the EC_50_ of propofol required for LMA-Supreme™ insertion was 2.50 ± 0.80 μg.mL^−1^ (95 % CI, 1.82–3.17 μg.mL^−1^; *p* < 0.0001).

**Fig. 4:**
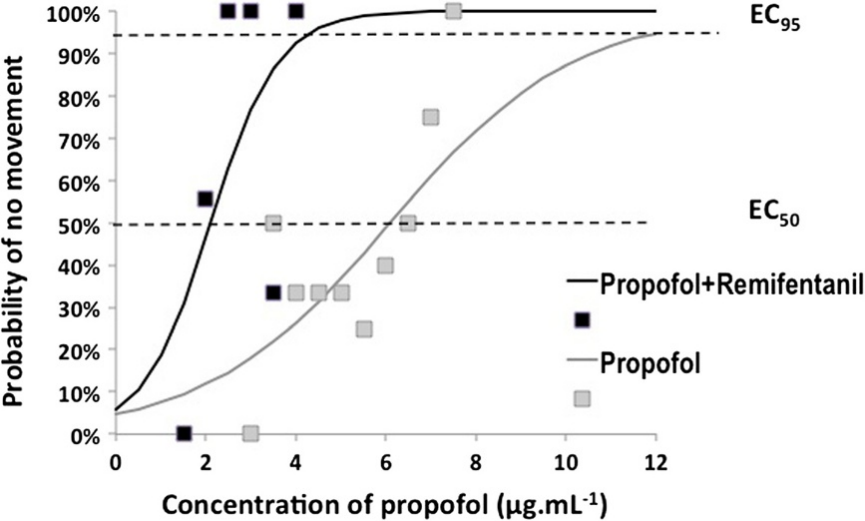
Dose–response curves plotted from logistic analysis of individual propofol concentrations illustrating reactions to LMA-Supreme™ insertion. EC_50_ in propofol + saline group: 6.09 μg.mL^−1^; EC_50_ in propofol + remifentanil group: 2.11 μg.mL^−1^; EC_95_ in propofol + saline group: 12.09 μg.mL^−1^; EC_95_ in propofol + remifentanil group: 4.33 μg.mL^−1^

Using logistic regression curves, the probability of no movement relative to the propofol concentration was determined in both groups (Fig. 4). The EC_50_ and EC_95_ values were 6.09 (95 % CI, 4.41–8.42 μg.mL^−1^) and 12.09 (95 % CI, 4.73–30.9 μg.mL^−1^), respectively, in the propofol + saline group, versus 2.11 (95 % CI, 1.19–3.72 μg.mL^−1^) and 4.33 (95 % CI, 1.37–13.6 μg.mL^−1^), respectively, in the propofol + remifentanil group. Table 2 presents the estimated values from the logistic and goodness of fit analyses.

**Table 2:** Estimated values of the logit coefficients

	Propofol + saline	Propofol + remifentanil
EC_50_ LMA-Supreme (CI)	6.09 (4.41–8.42 μg.mL^−1^)	2.11 (1.19–3.72 μg.mL^−1^)
EC_95_ LMA-Supreme (CI)	12.09 (4.73–30.9 μg.mL^−1^)	4.33 (1.37–13.6 μg.mL^−1^)
B0	−2.992	−2.794
B1	0.491	1.326
P-value	0.946	0.010
Goodness of fit chi-squared	2.81	13.24

CI: 95 % confidence interval

p/(1 − p) = B_0_ + B_1X_

B0 = intercept; B1 = slope; X = dose of propofol (μg.mL^−1^)

In seven patients (three in the propofol + saline group and four in the propofol + remifentanil group), we exchanged the LMA-Supreme™ for a LMA-ProSeal™ because of inadequate ventilation. All of these patients exhibited movement after LMA-Supreme™ insertion.

Baseline BIS and haemodynamic data did not differ between the two groups (Table 3). BIS values, systolic blood pressure, and diastolic blood pressure decreased throughout the study period in both groups. Prior to and 1 min after LMA-Supreme™ insertion, systolic and diastolic blood pressure decreased significantly more in the propofol + remifentanil group relative to the propofol + saline group. However, at 6 min after LMA-Supreme™ insertion, there were no significant differences in systolic and diastolic pressures between the groups.

**Table 3:** Bispectral index and haemodynamic data in the two study groups

Variable		Propofol + saline	Propofol + remifentanil
		(*n* = 32) [Mean % difference from baseline]	(*n* = 26) [Mean % difference from baseline]
BIS	Baseline	96 (3)	97 (2)
	Before SLMA insertion*^,^ **	36 (12) [63 %]	53 (14) [45 %]
	1 min after SLMA insertion*^,^ **	37 (15) [64 %]	51 (15) [47 %]
	6 min after SLMA insertion*^,^ **	28 (9) [71 %]	42 (16) [57 %]
Systolic BP (mmHg)	Baseline	125 (15)	131 (15)
	Before SLMA insertion*^,^ **	108 (13) [14 %]	95 (15) [27 %]
	1 min after SLMA insertion*^,^ **	111 (17) [11 %]	94 (15) [27 %]
	6 min after SLMA insertion*	97 (14) [23 %]	92 (11) [29 %]
Diastolic BP (mmHg)	Baseline	76 (10)	78 (12)
	Before SLMA insertion*^,^ **	66 (11) [12 %]	54 (12) [29 %]
	1 min after SLMA insertion*^,^ **	68 (11) [12 %]	56 (12) [29 %]
	6 min after SLMA insertion*	56 (11) [26 %]	55 (9) [28 %]
Heart rate (bpm)	Baseline	72 (18)	71 (10)
	Before SLMA insertion*^,^ **	71 (8) [0.01 %]	58 (9) [18 %]
	1 min after SLMA insertion*^,^ **	74 (12) [0,03 %]	58 (10) [18 %]
	6 min after SLMA insertion*^,^ **	62 (10) [14 %]	54 (8) [24 %]

Data are expressed as mean (SD)

*BIS* bispectral index, *BP* blood pressure, *SD* standard deviation

*p < 0.05; significant difference from baseline (difference within the group)

**p < 0.05; significant difference between the propofol + saline and propofol + remifentanil groups by ANOVA with repeated measurements

Heart rate significantly decreased relative to baseline values in both groups; notably, heart rate remained statistically lower in the propofol + remifentanil group compared with the propofol + saline group throughout the study period. The number of patients who required vasopressor or atropine administration for hypotension or bradycardia (three in each group) did not statistically differ between the groups.

BIS values decreased from baseline in both groups; however, after induction, values were statistically lower in the propofol + saline group compared with the propofol + remifentanil group (Table 3). None of the patients manifested recall of intraoperative events after anaesthesia.

Patient responsiveness to LMA-Supreme™ insertion did not significantly differ between the two groups (Table 4). Desaturation to less than 90 % during the anaesthetic induction and LMA-Supreme™ insertion occurred in two patients, both belonging to the propofol + remifentanil group.

**Table 4:** Assessment of jaw relaxation according to Muzi score

	Propofol + saline	Propofol + remifentanil
	*n* = 32	*n* = 26
Fully relaxed.	22	13
Mild resistance.	4	4
Resistance but could be opened.	2	3
Resistance requiring a dose of propofol.	4	6

Data regarding airway leak pressure and fibreoptic position of the airway tube are provided in Table 5.

**Table 5:** Oropharyngeal leak pressure and fibreoptic position of the airway tube

	Propofol + saline	Propofol + remifentanil
	(*n* = 32)	(*n* = 26)
Oropharyngeal leak pressure (cm H2O)	22 (3) [21–23]	26 (6) [23–29]*
Fibreoptic position 4/3/2/1 n (%)	11(39)/1(4)/4(14)/12(43)	6(26)/3(13)/8(35)/6(26)

Data are mean (SD) [95 % CI] or percentages

The fibreoptic position was available in 28 patient in propofol + saline group and in 23 patients in propofol + remifentanil group

1 = vocal cords not seen; 2 = vocal cords plus anterior epiglottis seen; 3 = vocal cords plus posterior epiglottis seen; 4 = only vocal cords visible

*p < 0.05 by unpaired Student’s *t*-test

## Discussion

This study determined the EC_50_ of propofol required for LMA-Supreme™ insertion with and without remifentanil at 5 ng.mL^−1^. We found that the addition of remifentanil reduced the EC_50_ of propofol necessary for LMA-Supreme™ insertion by 60 %. This is in accordance with previous reports, which have demonstrated that remifentanil reduces the dose of propofol required to blunt patients’ responses to different noxious stimuli as a consequence of the pharmacodynamic interactions of propofol and remifentanil [17].

Several studies have been previously conducted to determine the optimal effect-site concentration of propofol required for successful supraglottic device insertion, but most have evaluated the propofol dose required for Laryngeal Mask Airway Classic™ (LMA-Classic™) insertion. In these studies, the reported EC_50_ of propofol needed for LMA-Classic™ insertion ranged from 3.14 to 7.30 μg.mL^−1^ [18–20]. This variation may be related to confounding variables associated with the design of these studies, such as premedication, the use of co-induction agents, and the employment of different criteria to evaluate the success of insertion.

In our study, in the absence of remifentanil, the effect-site propofol concentration required to insert the LMA-Supreme™ in 50 % of patients was 6.32 μg.mL^−1^, which falls in the middle of the range described for the LMA-Classic™. In comparison with other sophisticated supraglottic devices, our required doses were higher than those described for the LMA-ProSeal™ (from 4.32 to 4.94 μg.mL^−1^) [18]. Given the design of the LMA-Supreme™, we anticipated that the EC_50_ of propofol necessary for insertion would be similar to that described for the LMA-ProSeal™. However, in a 2013 study of 31 adults, Zaballos and colleagues found that sevoflurane requirements for LMA-Supreme™ insertion were slightly higher than the concentrations required for LMA-ProSeal™ placement (3.03 % versus 2.82 %, respectively) [21]. This difference might be attributed to the use of co-induction agents that reduce patients’ responses to LMA insertion during propofol induction (higher doses of midazolam and lidocaine were used in the LMA-ProSeal™ study) [18, 22, 23] or to structural differences between the two devices resulting in unique mucosal pressures and different anaesthetic requirements.

Opioids have been extensively used as adjuvants to reduce the amount of propofol required to insert different supraglottic devices. Remifentanil is a potent opioid, with the relevant advantages of having a faster onset of action, a predictable offset of action, and not inducing prolonged respiratory depression [4]. Two reports found that remifentanil boluses of 0.25 and 0.50 μg.kg^−1^ improved LMA-Classic™ insertion conditions while maintaining haemodynamic stability when administered concurrently with propofol; however, remifentanil at 0.5 μg.kg^−1^ resulted in significant haemodynamic disturbances [24, 25].

Remifentanil has pharmacokinetic and pharmacodynamic properties that make it an ideal agent to use with TCI because it maintains a target opioid concentration at the effect site with a high degree of accuracy and minimal adverse haemodynamic changes [4]. Several reports have investigated the EC_50_ of remifentanil when used with a constant TCI of propofol.

In a study of 20 patients (who were premedicated with 40 mg lidocaine) that employed the up-and-down method, Kim MK and colleagues found that the EC_50_ of remifentanil required for LMA-Classic™ insertion was 3.04 ng.mL^−1^ in association with a TCI of 3.5 μg.mL^−1^ propofol. These values are similar to our data, although we used a higher dose of remifentanil (5 ng.mL^−1^ instead of 3.04 ng.mL^−1^) and, consequently, the EC_50_ of propofol was 2.5 μg.mL^−1^ in our study versus 3.5 μg.mL^−1^ in Kim’s report [8].

In a posterior study, Kim SH and colleagues compared the effect-site concentration of remifentanil required for Streamlined Liner of the Pharynx Airway (SLIPA™) and Laryngeal Mask Airway SoftSeal (LMA SoftSeal ™) insertion in adults during a TCI of 3.5 μg.mL^−1^ propofol. The authors observed that the EC_50_ values of remifentanil for SLIPA™ and LMA SoftSeal insertion were 0.93 and 1.36 ng.mL^−1^, respectively [26]. This combination of remifentanil and propofol yielded lower EC_50_ values than the authors had previously reported for the LMA-Classic™. This discrepancy may be attributed to differences in the anaesthetic technique used; in the second comparative study mentioned, a combination of 0.05 mg.kg^−1^ midazolam and 1 mg.kg^−1^ lidocaine was administrated before induction, whereas in the former study only 40 mg lidocaine was co-administered with propofol. Several previous studies have described the effects of midazolam and lidocaine in decreasing propofol requirements for LMA-Classic™ insertion [22, 23]. In our study, we administrated a fixed midazolam dose of 1 mg without lidocaine; consequently, the EC_50_ of propofol obtained was higher compared with previous reports. However, we cannot exclude from consideration the possibility that structural differences between the LMA-Classic™ and LMA-Supreme™ demand the application of different forces within the oropharyngeal structures and thereby necessitate different levels of anaesthesia.

In children, the addition of 7 ng.mL^−1^ of remifentanil reduced the EC_50_ of propofol required for successful insertion of the LMA-Classic™ (from 5.45 to 2.57 μg.mL^−1^) and a laryngeal tube (from 5.58 to 2.59 μg.mL^−1^). We observed a similar reduction in propofol concentration requirements in our study (from 6.32 to 2.50 μg.mL^−1^) with a lower remifentanil dose (5 ng.mL^−1^) [27]. This dissimilarity may be explained by pharmacokinetic and pharmacodynamic variations between paediatric and adult populations.

During the study, patients’ haemodynamic responses were similar in both groups with the exception of heart rate, which was strongly affected by remifentanil (Table 2). This is not an unexpected finding, and it is consistent with previous reports, which have revealed that the use of remifentanil is associated with more frequent episodes of bradycardia compared with other opioids [28].

An interesting finding in our study was that the OLP was higher (up to 18 % higher) in the propofol + remifentanil group compared with the propofol + saline group. The depth of anaesthesia, degree of muscle relaxation, cuff volume, and intracuff pressure are all factors that affect the seal between the respiratory tract and supraglottic devices [29]. However, the intracuff pressure was identical in all patients, and patients in the propofol + saline group had lower BIS values than patients in the propofol + remifentanil group (Table 2). In principle, the depth of anaesthesia could influence the seal, although there are no studies directly addressing this issue. The significant difference in the OLP between the two groups suggests that the combination of propofol and remifentanil results in greater suppression of the pharyngeal reflexes and possibly the tone of the pharyngeal muscles as well, thereby enabling a superior seal in the propofol + remifentanil group.

### Study limitations

In this study, we could have used a lower step size for propofol (less than 0.5 μg.mL^−1^), which would have provided greater accuracy [18–20]. However, the 0.5 μg.mL^−1^ step size is the common increment used in studies evaluating the EC_50_ of propofol required to insert supraglottic devices, and this consistent approach allowed us to perform comparisons. Moreover, if the step size had been reduced, more subjects would have been required to conduct the trial.

Although our data showed large inter-individual variability (Fig. 4), several aspects of our study support the accuracy of our results. Importantly, the starting propofol dose was close to that reported by other studies. This allowed us to avoid using a markedly different dose from the true dose, which would have increased the number of patients required to achieve the predetermined numbers of crossovers. We also increased the number of crossovers to seven (instead of the four crossovers suggested by Dixon’s original design) to improve the accuracy of our data.

In this study, the size of the LMA-Supreme™ was principally selected based on the sex of the patient (size 4 for females, size 5 for males) instead of the manufacturer’s weight-based suggestions. We do not know whether this approach could have changed the EC_50_ of propofol obtained in our study. However, sex-based LMA-Supreme™ selection is commonly used in investigations and probably in the clinical scenario as well [2, 3].

Next, the time needed to obtain equilibration of the propofol concentrations was 10 min in this study; this long time is not representative of usual clinical practice. However, our study aimed to investigate the EC_50_ of propofol required to insert the LMA-Supreme™ according to a predetermined methodology, the sequential up-and-down design. This design, similar to minimal alveolar concentration (MAC) determination, requires a minimum amount of time to ensure full propofol equilibration.

Finally, the EC_95_ is probably more relevant than the EC_50_ because this concentration represents anaesthetic requirements in clinical practice (patients need to be adequately anaesthetized to insert the supraglottic devices). However, the up-down method is designed to determine the EC_50_, and it is possible to estimate the EC_95_ by extrapolating on the logistic regression curve. In addition, prior key studies that investigated this topic evaluated the EC_50_ of anaesthetics required to insert airway devices. Thus, determination of the EC_50_ allowed us to compare our results with those of previous studies.

Finally, the haemodynamic responses observed in our study are applicable only to healthy patients aged 18–60 years. We cannot assume equivalent haemodynamic tolerance in more vulnerable ASA III or IV patients.

## Conclusions

Our study demonstrated that the propofol requirement for smooth insertion of the LMA-Supreme™ was 60 % less when 5 ng.mL^−1^ remifentanil was co-administered. Using the up-and-down method, we determined that the EC_50_ of propofol necessary for LMA-Supreme™ insertion was 2.50 ± 0.80 μg.mL^−1^ (95 % CI, 1.82–3.17 μg.mL^−1^) with remifentanil co-administration at 5 ng.mL^−1^ and 6.32 μg.mL^−1^ (95 % CI, 5.69–6.94 μg.mL^−1^) without concurrent remifentanil.

## Abbreviations

BIS: Bispectral index
MAC: Minimal alveolar concentration
EC_50_: Effect-site concentration in 50 %
EC95: Effect-site concentration in 95 %
LMA: Laryngeal mask airway
OLP: Oropharyngeal leak pressure
TCI: Target controlled infusion
